# Achieving Equitable Access to Medicines and Health Services: A COVID-19-time Recalled Matter

**DOI:** 10.22037/ijpr.2021.116120.15709

**Published:** 2021

**Authors:** Taraneh Mousavi, Shekoufeh Nikfar, Mohammad Abdollahi

**Affiliations:** a *Toxicology and Diseases Group (TDG), Pharmaceutical Sciences Research Center (PSRC), The Institute of Pharmaceutical Sciences (TIPS), Tehran University of Medical Sciences, Tehran, Iran. *; b *Department of Toxicology and Pharmacology, School of Pharmacy, Tehran University of Medical Sciences, Tehran, Iran. *; c *Evidence-Based Evaluation of Cost-Effectiveness and Clinical Outcomes Group, Pharmaceutical Sciences Research Center (PSRC), and the Pharmaceutical Management and Economics Research Center (PMERC), The Institute of Pharmaceutical Sciences (TIPS), Tehran University of Medical Sciences, Tehran, Iran. *; d *Department of Pharmacoeconomics and Pharmaceutical Administration, School of Pharmacy, Tehran University of Medical Sciences, Tehran, Iran.*

**Keywords:** COVID-19, Equity, Health, Inequity, Medicine, Pharmaceutical, Policy

## Abstract

In the 21^st^ century, while some people seek to use artificial intelligence for health services delivery, others have to surrender their health rights to meet basic needs. The gradient in health has become more pronounced in the COVID-19 crisis considering discrepancies in disease prevalence, geographical accessibility, availability, affordability, quality/safety of health services, and human resources. Through PubMed, GoogleScholar, Scopus, WHO, OECD, and UN databases, the English documents and global statistics were collected. Determining the role of health equity-related factors and introducing mechanisms to maintain regional and international justice in health, specifically during the COVID-19 pandemic, were among the core concepts of this paper. Social determinants of health (SDH), interregional and intraregional bodies are the main drivers of discrimination in health services. Governments should relish chief health strategists’ role in possessing legitimacy, accountability, direction, transparent performance, fairness, and good governance in one word. Improving health literacy and telemedicine, providing income support, and reforming insurance where needed, are other national mechanisms to amend inequity. Among interregional issues, what is concerning is the matter of sanctions on access to health services, which is against the Universal Declaration of Human Rights. Shortage of vital medications, ventilators, test kits, COVID-19 vaccines, pharmaceutical raw materials, foreign currency, decreased national currency value, purchasing power parity, and quality/safety of health services resulted from such oppression. The article also provides practical suggestions, paving the way for re-establishing global solidarity and developing health justice in deprived regions.

## Introduction

In the 21^st^ century, while people in some countries seek to use artificial intelligence for health services delivery, others have to abdicate their health’s right to meet basic life needs like housing and food (1) Reports have indicated that in 2015, the percentage of total population pushed below the USD 1.90 a day poverty line by household health expenditures were 1.3%, 0.003%, 0.23%, 0.021%, and 1.66% in Africa, Northern America, Latin America, the Caribbean, Europe, and Asia, respectively (2). This so-called “global inequity crisis” is primarily against the universal declaration of human rights provided by the United Nations (UN) and World Health Organization (WHO) in 1948 (3, 4). It emphasizes that timely access to physical and mental health care services at the highest quality is a fundamental human right that should not be affected by social determinants of health (SDH), *i.e*., gender, age, ethnicity, education level, economic status, career, and place of residence (5). Put it differently, availability, affordability, quality/safety, and geographical accessibility to health services and human resources should not become barriers endangering people’s life (3, 6). Unfortunately, the opposite of these slogans is seen in today’s world. In health care services, the mentioned determinants of accessibility and quality, if not fully observed, lead to poor treatment adherence or even discontinuation of the treatment; thereby, worsening disease (3). On the other side, high cost or shortage in diagnostic/preventive methods, especially in life-threatening illnesses (e.g., cancer or transmissible conditions), affect many individuals. In some situations, the lack of novel diagnostic procedures forces clinicians to use invasive approaches causing patients to suffer. One of the examples of this is the absence of capsule endoscopy devices as an alternative to conventional imaging methods. The capsule endoscopy platform uses innovative visualization techniques to produce clear images of the esophagus, stomach, small bowel, and colon. This technique is not invasive and may have more patient compliance than conventional endoscopy procedures. 

The overall results of all these are increased incidence, prevalence, and economic burden of a specific disease and reduced life expectancy (7, 8). Indeed, the unfair allocation of health-related resources around the globe creates a vicious cycle. Due to some underlying factors, including but not limited to high inflation, low income, and residence place, people cannot meet their basic health needs and unconsciously endure mental pressure (9). These issues give rise to the progression of their pre-existing diseases and the emergence of new problems, mainly stress-derived and mental disorders, imposing additional costs on patients and their relatives. Higher rates of morbidity and mortality due to reproductive issues, non-communicable diseases (NCDs), infectious diseases, heredity, and deficiency diseases in low- and middle-income countries are compelling evidence supporting the unmet need of the affected patients (10-12). Excessive alcohol dependence/drug addiction and consequently associated disorders in poor regions are mainly rooted in a lack of efficient withdrawal mechanisms/treatments and undue economic pressure (3). Despite prior claims concerning fundamental human rights and strategies like universal health coverage (UHC)(8, 13), it seems that there has been an insufficient concentration on decisive factors related to the “social gradient in health” over past decades. Comparison of the profound impacts of these factors on health equity and introducing mechanisms to maintain regional and international justice in health, specifically during the Coronavirus disease of 2019 (COVID-19) pandemic, were among the core concepts of this paper.

**Search strategy**

This mini-review was provided through accessing major electronic databases, *i.e*., PubMed, Google Scholar, and Scopus. Over the past 5 years, a comprehensive search was done on key terms, including but not limited to, “equity”, “equality”, “inequity”, “inequality”, and “sanctions” together with “health”, “science”, “education”, “device”, and “instrument”. WHO, Organization for Economic Co-operation and Development (OECD), and United Nations (UN) databases were studied to extract guidelines and statistics related to SDH and health equity. 

**Main issues responsible for health inequity**

**Figure 1:**
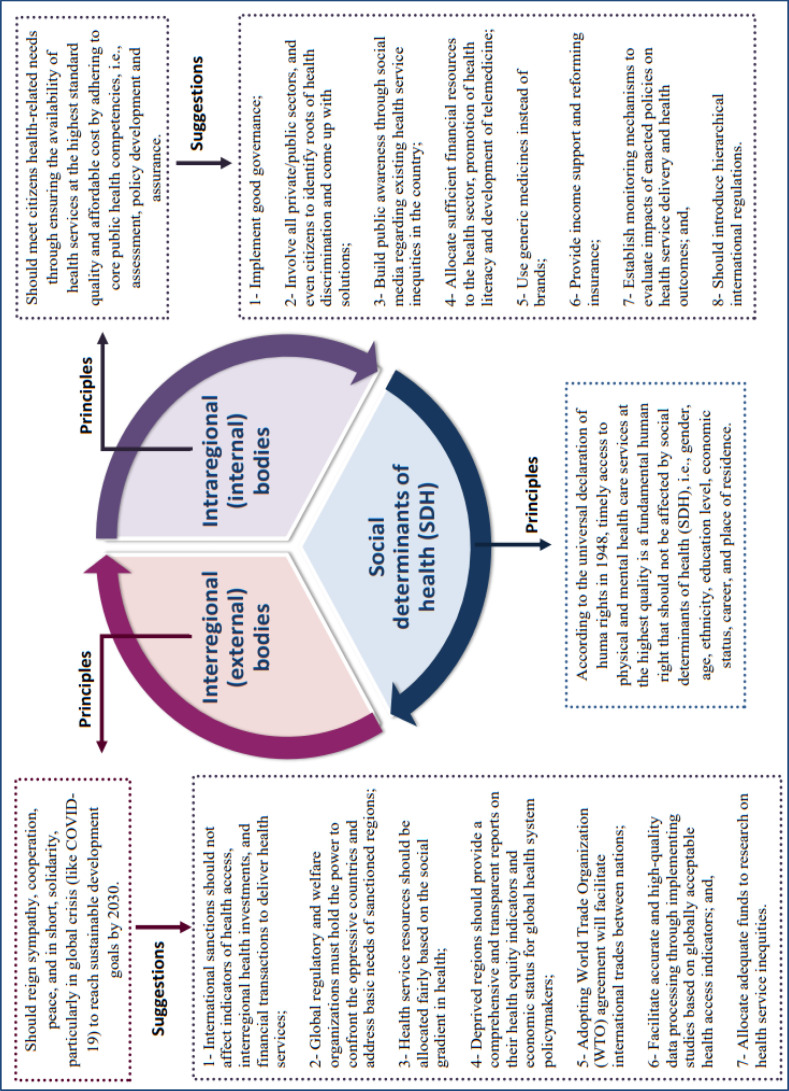
Leading factors affecting equity in health services and recommended mechanisms to improve their role

In general, three main issues are at play: 1) internal or intraregional bodies; 2) external or interregional bodies; and 3) SDH (10, 14). As shown in Figure 1, there is a tripartite relationship among these factors, which has been considerably disrupted during the recent crisis of COVID-19. 

*Internal bodies and SDH*

The role of internal or intraregional bodies makes sense in meeting citizens’ health-related needs through ensuring the availability of health services at the highest standard quality and affordable cost by adhering to core public health competencies, *i.e*., assessment, policy development, and assurance (15). More broadly, the sufficient health sector should be able to reduce health inequity through providing appropriate living and working conditions, improving social and human capacity, and strengthening income security and social protection (7, 10). To this end, the government should principally relish chief health strategists’ role in possessing legitimacy, accountability, direction, transparent performance, fairness, and good governance in one word (9, 10, and 15). Nonetheless, exclusive health system management by a limited number of people has no result other than a fragile system full of flaws and inequities (10). In the modern era of collaboration, it is suggested to involve all related private and public sectors and even citizens to identify roots of health discrimination and come up with solutions (9, 10, 13, and 15). Building public awareness through social media regarding existing health service inequities is another practical approach to engaging the younger generation with innovative resolutions (9). Since the direct relation of health care system investments with improvement in health services equity has been proved in most studies, internal bodies should allocate sufficient financial resources to the health sector, promote health literacy, and develop telemedicine (1, 10, 16, and 17). An example of this can be achieved by comparing countries’ data in Table 1. Albeit, what is critical is a balanced investment in all sectors so that people’s health is not affected by SDH. In other words, there should be mechanisms to lessen disease incidence and consequently the public need for medications and diagnostic systems. Lack of proper investment in personalized medicine and gene therapeutics in underdeveloped and developing countries is another issue that renounces the right to adopt personalized approaches with better efficacy and safety in clinical settings. Insufficient investments in health literacy may cause the latest medical and surgical techniques not to be applied worldwide. In countries with poor accessibility to essential medications, replacing generics instead of brands would benefit. However, there must be tight regulations to warrant the efficacy/safety of generics substitutes (6). Based on the economic status and insurance coverage, in some regions, providing income support and reforming insurance is warranted to reduce the proportion of uncovered people and out-of-pocket expenses, especially in conditions like the current pandemic (8, 16, and 18). Following determination of the roots of internal injustice (assessment) and legislation for providing accessible health services with good quality (policy development), assurance should be obtained through establishing monitoring mechanisms to evaluate impacts of enacted policies on the state of health inequalities in terms of health service delivery and health outcomes (13). Intraregional bodies should ensure that health services allocation is not affected by SDH within the abovementioned activities. Shortage of health care providers, personal protective equipment, laboratory facilities, intensive care units, and diagnostic and therapeutic interventions in the rural areas are not justifiable considering fundamental human rights.

Another example is that some ethnic minority groups are not involved in many global clinical trials. Therefore, the right to determine the medications’ efficacy and side effects are taken away (11, 19, and 20). For instance, a study from July 2008 to June 2018 on all clinical trials related to USFDA oncology medication approvals revealed that despite a growing burden of cancer among all ethnicities, racial minorities, including Blacks and Hispanics, have been overlooked in these clinical trials recruitment (20, 21). Another similar study between 2003 and 2016 on cancer clinical trials also indicated a reduction in enrollment of patients from African American and Hispanic ethnic groups (22).

*External bodies*

Even with the most efficient and transparent health system, the adverse impacts of external bodies in provoking health services inequity cannot be overlooked. The issue of international sanctions on health services is the most catastrophic effect, thoroughly against the Universal Declaration of Human Rights of 1948 and the International Covenant on Economic, Social, and Cultural Rights of 1966 (3, 4). Strict sanctions imposed on Iraq, Cuba, Haiti, Syria, and worst of all, Iran have limited their access to diagnostic, preventive, and treatment interventions for decades (3, 23). The significance of this matter is that about 44% of medications in the WHO list of essential medicines have not been available in Iran (3). Access to some nuclear and radiotherapy services for cancer has also been banned (3). Since the pharmaceutical sector of Iran is dependent on obtaining raw materials from foreign countries, the country has faced significant difficulties due to a lack of financial transactions (3, 4, 24). Inevitably, raw materials have to be acquired from cheap and new sources affecting the final quality of medicines. Moreover, the number of expired and contaminated smuggled drugs with lower quality may rise (3). Rather than directly influencing health services, a threat of sanctions can markedly shake the economic status by reducing the national currency value, shortage of foreign currency, and household income, all of which lessen purchasing power parity (23).

*The crisis of Coronavirus disease-2019 (COVID-19) pandemic*

**Figure 2:**
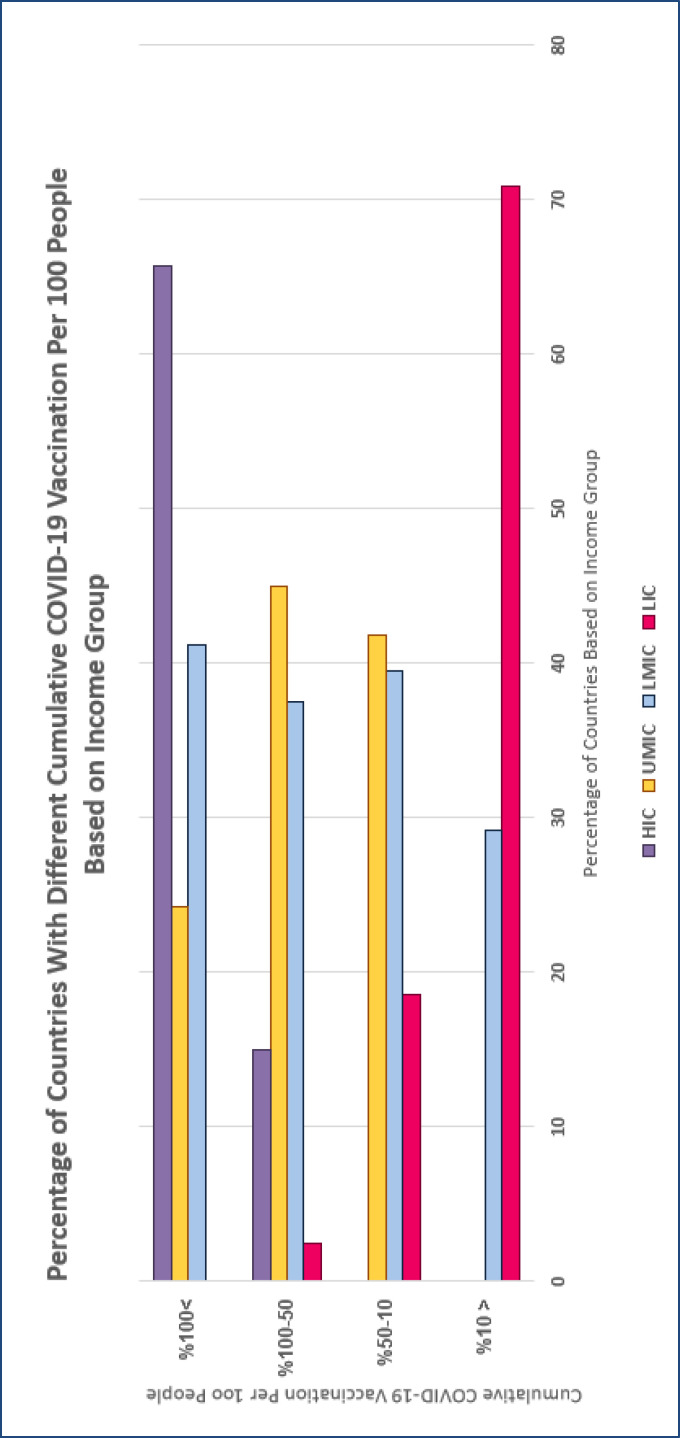
Percentage of countries with different cumulative (<10%; 10-50%; 50-100%; > 100%) COVID-19 vaccination per 100 people based on income group, up to 17 November 2021. Abbreviations: HIC: High-income country; LIC: Low-income country; LMIC: Lower middle-income country; UMIC: Upper middle-income country

A crisis like the COVID-19 pandemic can itself exacerbate health injustice since it is associated with a reduction in household income, job loss, increased out-of-pocket expenses, lack of equitable access to telemedicine, exacerbation of pre-existing illnesses, and a further rise in health care costs, particularly among elderly, socially excluded people and ethnic minority groups (13, 19, 24, 25). For instance, inaccessibility to telemedicine in the majority of countries has provoked COVID-19-unrelated mortalities or pre-existing disease worsening, especially at the beginning of the pandemic, since people with chronic disease fear to follow up their condition, refuse routine clinician visits, and even do regular monitoring like complete blood count (CBC) test. What is most striking during this pandemic is an overt gap in access to diagnostic test kits, knowledge, emergency medical equipment, pharmaceuticals, and more to the point, vaccines. Up to November 17, 2021, 3.61 billion doses were administered by upper-middle-income countries, followed by 2.18 billion doses by lower-middle-income, 1.75 billion doses for high income, and 43.69 million doses by low-income countries (26). Look it differently, as it is shown in Table 1 and Figure 2, most of the countries (70.83%) for which the cumulative COVID-19 vaccination per 100 people is less than 10% are from low-income groups.

**Figure 3:**
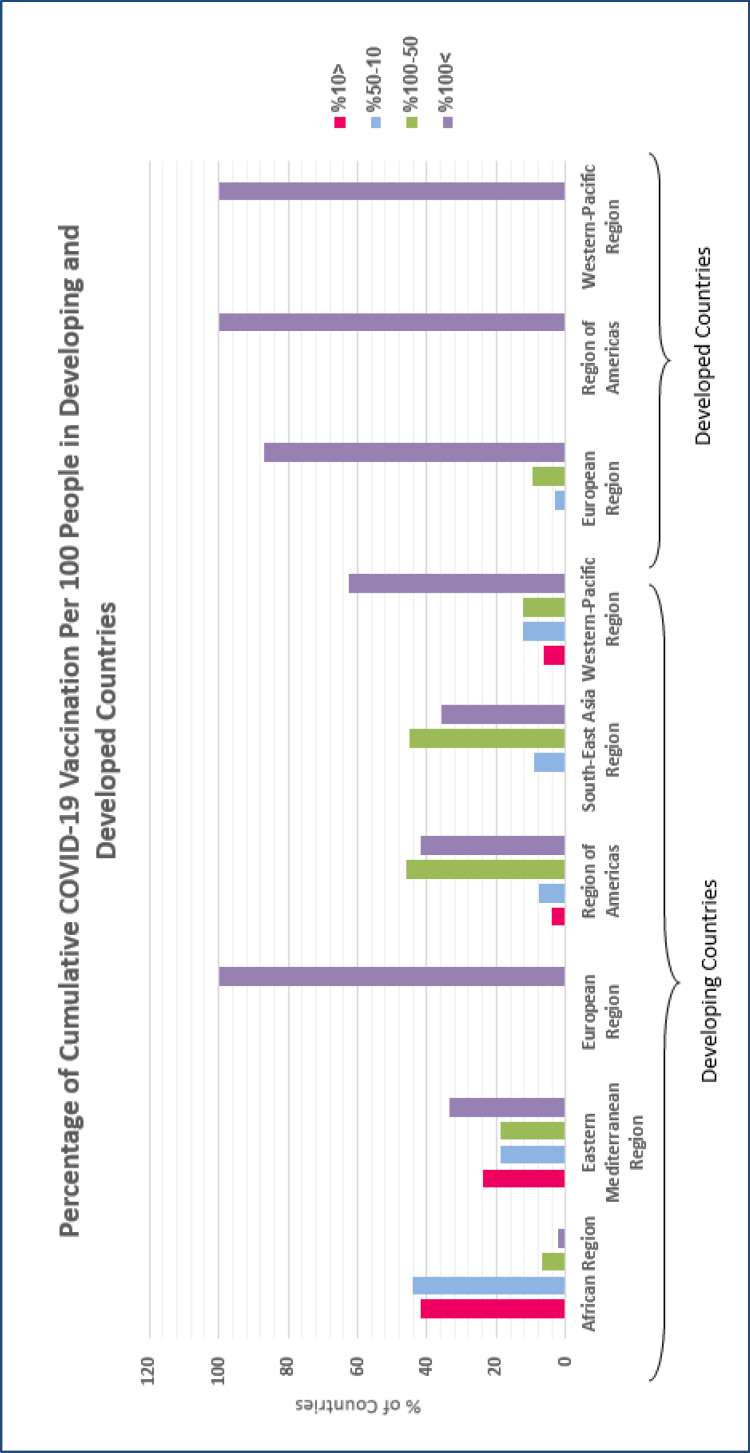
Percentage of cumulative COVID-19 vaccination per 100 people in developing and developed countries, up to 17 November 2021

On the contrary, the cumulative COVID-19 vaccination per 100 people in all high-income regions is above 50%. Such inequity can be justified considering fragile health systems, lack of interregional solidarity, and the high price of vaccines for many low- and middle-income regions (27). Moreover, there is an absolute relation between the level of a country’s development and the amount of vaccination. As it is summarized in Table 1 and Figure 3, COVID-19 vaccination in developed regions has almost covered all of the population. However, developing countries still have deficits, particularly in African and Eastern Mediterranean WHO regions. 

Designing legislation on UHC, *i.e*., “all people have access to the health services they need, when and where they need them, without financial hardship”, is another factor generally in favor of COVID-19 vaccination and better access to health-related services equipment. According to Table 1, about 56.07% of regions with more than 50% cumulative COVID-19 vaccination per 100 people have adapted UHC. This is while 86.88% of countries without legislation on UHC have less than 50% of cumulative COVID-19 vaccination per 100 people. 

Apart from vaccination, the COVID-19 pandemic has created inequities in the concept of “international distribution” worldwide. Namely, due to fear of shortage in resources and medical equipment needed to manage the pandemic internally, some countries banned pharmaceutical raw materials and other medical equipment exportation, mainly ventilators. On the other hand, many regions have refused world trade and navigation as they are afraid of the COVID-19 prevalence through transportation routes. These issues have caused some low- and middle-income countries (4), even those with the active pharmaceutical industry and COVID-19 vaccine in their pipeline, to face problems during the production of domestic COVID-19 vaccines and generic medications since they are firmly dependent on the import of raw materials (24).

Rather than the direct effects of low vaccination rate on the total mortality from COVID-19, it can influence countries’ economic recovery post-COVID-19 pandemic, *i.e*., countries with a lower rate of vaccinations are expected to have minor growth in the annual gross domestic product (GDP) and difficulties in reaching pre-COVID-19 economic and developmental status by the next five years (27, 28). 

Noteworthy, in the context of sanctioned countries, absence of prior national investments in the field of vaccine manufacturing, US threat to impose sanctions on countries under collaboration with Iran, lack of financial transactions and cooperation with multinational pharmaceutical companies, high demand and instant cash payments from powerful governments, and shipment limitation has made the mentioned condition catastrophic (4). Lower rate of COVID-19 vaccination in sanctioned countries and consistent high COVID-19 morbidity and mortality compared to other regions are not, therefore, far from expectation. The consequence of such injustice will eventually affect the whole global population; given migration and international transportation, a lower number of vaccinated individuals is associated with advanced risk of the COVID-19 prevalence and emergence of new, probably more life-threatening, mutations. 

Despite all these, when comparing vaccination data from August to November (Table 1), there is an about 2-fold increase in cumulative COVID-19 vaccination per 100 people. Primarily, it might be due to vaccine resources transfer from developed countries to deprived regions, when most of the population in developed countries has been fully vaccinated before November 2021 (Table 1). Another factor justifying such improvement is the allocation of national pharmaceuticals, and specifically biotechnological pipelines, for manufacturing COVID-19 vaccines. The fact that global authorities are accepting more types of COVID-19 vaccines and the approval of national COVID-19 vaccines in some regions could be other reasons. The latter is particularly true concerning Iran, where following the approval of COVIran Barekat and Spikogen, there has been a considerable rise in the vaccination rate. 

**Further suggestions**

In such a situation where many people die daily, other than national systems, global regulatory and welfare organizations must hold power to confront the oppressive countries and address the basic needs of sanctioned regions. They should also certify that health service resources should be allocated relatively based on the social gradient in health. To this end, deprived areas should provide a comprehensive and transparent report on their health equity indicators and economic status for global health system policymakers. This seems urgent considering that most of the available data on health equity is from WHO European regions. Such reports can be transferred through WHO regional offices and further be discussed in joint meetings. Adopting the World Trade Organization (WTO) agreement will facilitate international trades between nations (29). Through introducing proper legislation, health ministries should find strategies to maintain supply-demand balance for investigational/ approved medications in the treatment of COVID-19 with concomitantly processing other labeled indications (24). Improving public knowledge and combating misinformation might be effective in this context. 

Two other current interregional issues are the lack of adequate global funding for research on health disparities (15, 20) and data for decision-making (13, 15, 30). It is of high necessity to obtain accurate and high-quality data by investigating the dimension of health inequities, their roots and impacts on health outcomes, and comparing intraregional reports; thereby, processing evidence-based decision-making. In this regard, implementing studies based on globally acceptable health access indicators is strongly supported (6). 

All in all, sustainable development goals (SDGs) could not be attained by 2030 unless sympathy, cooperation, peace, and in short, solidarity reign worldwide.

**Table 1:** Comparison of important health equity indicators among global countries based on WHO region, development status, and income group (31).

**WHO region (32)**	**Country**	**Income group (33)**	**Cumulative COVID-19 vaccination per 100 people (26)** **(10/08/2021) **	**Cumulative COVID-19 vaccination per 100 people (26)** **(17/11/2021)**	**Current health expenditure (CHE) as a percentage of gross domestic product (GDP) (%) (2018) (34)**	**Health purchasing power parities (national currency per USD) (2017) (35)**	**Passed legislation on universal health coverage (UHC) (2017)(36)**
**Developing countries**							
**African region**	Algeria	LMIC	9.45 (29/07/2021)	25.5 (13/11/2021)	6.22	NA	Yes
	Angola ^¶^	LMIC	5.16 (09/08/2021)	23.01 (11/11/2021)	2.55	NA	No
	Benin ^¶^	LMIC	0.58 (03/08/2021)	2.79 (08/11/2021)	2.49	NA	No
	Botswana	UMIC	16.00 (06/08/2021)	47.06 (10/11/2021)	5.85	NA	Yes
	Burkina Faso ^¶^	LIC	0.18 (09/08/2021)	3.08 (04/11/2021)	5.63	NA	No
	Burundi ^¶^	LIC	NA	0.01 (12/11/2021)	7.74	NA	No
	Cape Verde	LMIC	36.83 (09/08/2021)	93.77 (10/11/2021)	5.36	NA	No
	Cameroon	LMIC	1.39 (09/08/2021)	1.91 (12/11/2021)	3.53	NA	No
	Central African Republic ^¶^	LIC	1.97 (05/08/2021)	8.58 (07/11/2021)	10.99	NA	No
	Chad ^¶^	LIC	0.22 (05/08/2021)	1.41 (10/11/2021)	4.10	NA	No
	Comoros ^¶^	LMIC	21.72 (09/08/2021)	53.62 (14/11/2021)	4.59	NA	No
	Congo ^¶^	LMIC	3.60 (29/07/2021)	9.71 (10/11/2021)	2.14	NA	Yes
	Côte d’Ivoire	LMIC	4.33 (08/08/2021)	13.63 (14/11/2021)	NA	NA	No
	Democratic Republic of the Congo	LIC	0.10 (09/08/2021)	0.18 (11/11/2021)	3.30	NA	No
	Equatorial Guinea	UMIC	22.40 (07/08/2021)	30.37 (15/11/2021)	3.00	NA	No
	Eritrea ^¶^	LIC	NA	NA	4.09	NA	No
	Eswatini	LMIC	10.29 (06/08/2021)	25.02 (11/11/2021)	6.54	NA	No
	Ethiopia ^¶^	LIC	1.99 (10/08/2021)	4.31 (16/11/2021)	3.30	NA	No
	Gabon Sao Tome and Principe ^¶ ^	LMIC	5.01 (09/08/2021)	49.11 (10/11/2021)	2.75	NA	No
	Gambia ^¶^	LIC	1.80 (15/07/2021)	10.76 (14/11/2021)	3.09	NA	Yes
	Ghana	LMIC	4.09 (19/07/2021)	10.05 (10/11/2021)	3.54	NA	No
	Guinea ^¶^	LIC	7.00 (06/08/2021)	17.52 (10/11/2021)	3.93	NA	No
	Guinea-Bissau ^¶^	LIC	1.55 (09/08/2021)	16.41 (15/11/2021)	7.00	NA	No
	Kenya	LMIC	3.34 (07/08/2021)	11.06 (15/11/2021)	5.17	NA	No
	Lesotho ^¶^	LMIC	3.41 (26/07/2021)	NA	9.28	NA	No
	Liberia ^¶^	LIC	1.89 (12/07/2021)	8.47 (02/11/2021)	6.74	NA	No
	Madagascar ^¶^	LIC	NA	1.99 (20/10/2021)	4.79	NA	No
	Malawi ^¶^	LIC	3.26 (08/08/2021)	6.73 (15/11/2021)	9.33	NA	No
	Mali ^¶^	LIC	1.28 (09/08/2021)	2.86 (02/11/2021)	3.88	NA	No
	Mauritania ^¶^	LMIC	5.09 (09/08/2021)	35.71 (15/11/2021)	4.58	NA	No
	Mauritius	HIC	101.99 (09/08/2021)	138.55 (10/11/2021)	5.83	NA	No
	Mozambique ^¶^	LIC	4.44 (09/08/2021)	23.19 (10/11/2021)	8.17	NA	No
	Namibia	UMIC	8.99 (08/08/2021)	23.74 (10/11/2021)	7.95	NA	No
	Niger ^¶^	LIC	1.76 (09/08/2021)	3.83 (14/11/2021)	7.33	NA	No
	Nigeria	LMIC	1.92 (09/08/2021)	4.27 (11/11/2021)	3.89	NA	No
	Rwanda ^¶^	LIC	6.59 (03/08/2021)	52.55 (11/11/2021)	7.54	NA	No
	Senegal ^¶^	LMIC	8.12 (09/08/2021)	11.40 (20/10/2021)	3.98	NA	No
	Sierra Leone ^¶^	LIC	NA	9.15 (10/11/2021)	16.06	NA	No
	South Africa	UMIC	14.50 (08/08/2021)	40.33 (16/11/2021)	8.25	NA	No
	South Sudan ^¶^	LIC	0.51 (19/07/2021)	1.33 (09/11/2021)	6.40	NA	No
	Togo ^¶^	LIC	5.73 (03/08/2021)	17.14 (12/11/2021)	6.17	NA	No
	Uganda ^¶^	LIC	2.53 (10/08/2021)	10.08 (15/11/2021)	6.53	NA	No
	United Republic of Tanzania ^¶^	LMIC	0.18 (08/08/2021)	1.63 (29/10/2021)	3.63	NA	No
	Zambia ^¶^	LMIC	2.74 (10/08/2021)	5.32 (17/11/2021)	4.93	NA	No
	Zimbabwe	LMIC	19.42 (08/08/2021)	41.27 (16/11/2021)	4.73	NA	No
**Eastern Mediterranean region**	Afghanistan ^¶ ^	LIC	4.54 (09/08/2021)	10.90 (14/11/2021)	9.4	NA	No
	Bahrain	HIC	140.35 (10/08/2021)	162.43 (17/11/2021)	4.13	NA	Yes
	Djibouti ^¶^	LMIC	5.11 (05/08/2021)	9.19 (03/11/2021)	2.32	NA	No
	Egypt	LMIC	5.52 (05/08/2021)	32.29 (10/11/2021)	4.95	NA	No
	Iran (Islamic Republic of)	UMIC	14.75 (02/08/2021)	115.47 (13/11/2021)	8.66	NA	No
	Iraq	UMIC	5.23 (06/08/2021)	25.94 (15/11/2021)	4.11	NA	No
	Jordan	UMIC	55.10 (10/08/2021)	74.95 (16/11/2021)	7.79	NA	Yes
	Kuwait	HIC	NA	NA	5.00	NA	Yes
	Lebanon	UMIC	31.20 (10/08/2021)	51.43 (17/11/2021)	8.35	NA	No
	Libya	UMIC	11.12 (09/08/2021)	30.90 (14/11/2021)	NA	NA	No
	Morocco	LMIC	71.90 (10/08/2021)	129.66 (14/11/2021)	5.31	NA	No
	Oman	HIC	52.02 (09/08/2021)	111.47 (09/11/2021)	4.13	NA	Yes
	Pakistan	LMIC	17.98 (10/08/2021)	53.29 (17/11/2021)	3.20	NA	No
	Qatar	HIC	138.52 (09/08/2021)	167.03 (17/11/2021)	2.49	NA	No
	Saudi Arabia	HIC	87.74 (10/08/2021)	132.47 (17/11/2021)	6.36	NA	No
	Somalia ^¶^	LIC	1.76 (02/08/2021)	5.09 (13/11/2021)	NA	NA	No
	Sudan ^¶^	LIC	1.88 (09/08/2021)	3.70 (20/10/2021)	4.51	NA	No
	Syrian Arab Republic	LIC	NA	7.34 (15/11/2021)	NA	NA	No
	Tunisia	LMIC	28.71 (08/08/2021)	83.17 (16/11/2021)	7.29	NA	Yes
	United Arab Emirates	HIC	173.88 (10/08/2021)	215.68 (15/11/2021)	4.23	NA	Yes
	Yemen ^¶^	LIC	1.04 (27/07/2021)	2.45 (14/11/2021)	NA	NA	No
**European region**	Israel	HIC	137.25 (10/08/2021)	172.80 (17/11/2021)	7.52	4.73	No
	Turkey	UMIC	92.80 (10/08/2021)	139.79 (17/11/2021)	4.12	0.73	Yes
**Region of Americas**	Bahamas	HIC	28.08 (09/08/2021)	66.46 (05/11/2021)	6.25	NA	No
	Belize	UMIC	48.93 (06/08/2021)	98.32 (12/11/2021)	5.69	NA	No
	Bolivia	LMIC	40.95 (09/08/2021)	67.96 (11/11/2021)	6.30	NA	Yes
	El Salvador	LMIC	74.91 (10/08/2021)	137.24 (16/11/2021)	7.11	NA	Yes
	Haiti ^¶^	LIC	0.14 (09/08/2021)	1.33 (12/11/2021)	7.69	NA	No
	Jamaica	UMIC	12.49 (06/08/2021)	35.60 (17/11/2021)	6.06	NA	No
	Argentina	UMIC	77.99 (10/08/2021)	142.45 (17/11/2021)	9.62	NA	Yes
	Barbados	HIC	62.47 (07/08/2021)	98.94 (17/11/2021)	6.56	NA	No
	Brazil	UMIC	73.12 (10/08/2021)	139.24 (17/11/2021)	9.51	NA	Yes
	Chile	HIC	137.50 (08/08/2021)	203.68 (14/11/2021)	9.14	416	Yes
	Colombia	UMIC	60.22 (09/08/2021)	101.34 (15/11/2021)	7.64	939	Yes
	Costa Rica	UMIC	71.52 (09/08/2021)	133.84 (15/11/2021)	7.56	535	Yes
	Cuba	UMIC	95.78 (08/08/2021)	243.59 (15/11/2021)	11.19	NA	Yes
	Dominica	UMIC	56.33 (30/08/2021)	74.76 (12/11/2021)	6.59	NA	No
	Dominican Republic	UMIC	96.79 (09/08/2021)	124.36 (16/11/2021)	5.73	NA	Yes
	Ecuador	UMIC	75.68 (08/08/2021)	129.15 (12/11/2021)	8.14	NA	Yes
	Guatemala	UMIC	15.50 (09/08/2021)	50.92 (16/11/2021)	5.71	NA	No
	Guyana	UMIC	52.90 (10/08/2021)	83.00 (16/11/2021)	5.94	NA	No
	Honduras	LMIC	21.67 (03/08/2021)	75.37 (12/11/2021)	7.05	NA	No
	Mexico	UMIC	56.61 (10/08/2021)	99.79 (17/11/2021)	5.37	10.4	No
	Nicaragua	LMIC	13.88 (09/08/2021)	24.99 (05/11/2021)	8.56	NA	No
	Panama	HIC	71.68 (07/08/2021)	126.21 (17/11/2021)	7.27	NA	Yes
	Paraguay	UMIC	29.78 (25/08/2021)	79.59 (12/11/2021)	6.65	NA	No
	Peru	UMIC	46.01 (09/08/2021)	113.23 (13/11/2021)	5.24	NA	No
	Suriname	UMIC	45.20 (10/08/2021)	79.01 (17/11/2021)	7.97	NA	No
	Trinidad and Tobago	HIC	47.73 (10/08/2021)	90.17 (17/11/2021)	6.93	NA	No
**South-East Asia region**	Bangladesh ^¶^	LMIC	11.71 (10/08/2021)	51.87 (17/11/2021)	2.34	NA	No
	Bhutan ^¶^	LMIC	130.91 (09/08/2021)	147.22 (31/10/2021)	3.06	NA	Yes
	Democratic People’s Republic of Korea	LIC	NA	NA	NA	NA	No
	India	LMIC	37.61 (10/08/2021)	81.89 (17/11/2021)	3.54	NA	No
	Indonesia	UMIC	28.13 (10/08/2021)	78.99 (17/11/2021)	2.87	NA	No
	Maldives	UMIC	113.74 (07/08/2021)	139.13 (15/11/2021)	9.41	NA	No
	Myanmar ^¶^	LMIC	NA	40.42 (06/11/2201)	4.79	NA	No
	Nepal ^¶^	LMIC	24.93 (10/08/2021)	54.50 (07/11/2021)	5.84	NA	No
	Sri Lanka	LMIC	64.28 (08/08/2021)	137.55 (17/11/2021)	3.76	NA	No
	Thailand	UMIC	30.33 (09/08/2021)	123.05 (17/11/2021)	3.79	NA	Yes
	Timor-Leste ^¶^	LMIC	30.56 (03/08/2021)	72.15 (09/11/2021)	4.33	NA	No
**Western Pacific region**	Brunei Darussalam	HIC	43.41 (08/08/2021)	158.45 (17/11/2021)	2.41	NA	Yes
	Cambodia ^¶^	LMIC	87.05 (10/08/2021)	167.16 (16/11/2021)	6.03	NA	No
	China	UMIC	125.62 (10/08/2021)	166.86 (17/11/2021)	5.35	NA	No
	Fiji	UMIC	77.07 (09/08/2021)	133.88 (15/11/2021)	3.42	NA	No
	Kiribati ^¶^	LMIC	11.70 (09/08/2021)	60.07 (15/11/2021)	12.11	NA	No
	Lao People’s Democratic Republic ^¶^	LMIC	NA	NA	2.25	NA	No
	Malaysia	UMIC	78.37 (10/08/2021)	156.71 (17/11/2021)	3.76	NA	No
	Mongolia	LMIC	128.46 (10/08/2021)	132.37 (16/11/2021)	3.79	NA	Yes
	Papua New Guinea	LMIC	1.12 (02/08/2021)	3.19 (25/10/2201)	2.37	NA	No
	Philippines	LMIC	22.81 (08/08/2021)	65.52 (17/11/2021)	4.40	NA	No
	Republic of Korea	HIC	55.72 (10/08/2021)	160.62 (17/11/2021)	7.56	NA	Yes
	Samoa	UMIC	50.71 (09/08/2021)	111.67 (15/11/2021)	5.21	NA	No
	Singapore	HIC	139.23 (09/08/2021)	184.89 (05/11/2021)	4.46	NA	Yes
	Solomon Islands ^¶^	LMIC	8.24 (09/08/2021)	24.37 (08/11/2021)	4.47	NA	No
	Vanuatu	LMIC	7.81 (27/07/2021)	39.07 (15/11/2021)	3.37	NA	No
	Viet Nam	LMIC	10.26 (09/08/2021)	103.93 (16/11/2021)	5.92	NA	No
**Developed countries**							
**European region**	Austria	HIC	111.47 (10/08/2021)	138.57 (17/11/2021)	10.33	0.96	Yes
	Belgium	HIC	131.70 (09/08/2021)	146.12 (16/11/2021)	10.32	0.94	Yes
	Bulgaria	UMIC	30.43 (10/08/2021)	45.39 (17/11/2021)	7.35	0.38	Yes
	Croatia	HIC	76.29 (09/08/2021)	95.53 (16/11/2021)	6.83	2.69	Yes
	Cyprus	HIC	115.73 (09/08/2021)	139.42 (16/11/2021)	6.77	0.78	Yes
	Czechia	HIC	100.64 (10/08/2021)	120.18 (17/11/2021)	7.65	7.60	Yes
	Denmark	HIC	134.50 (10/08/2021)	153.34 (16/11/2021)	10.07	6.83	Yes
	Estonia	HIC	89.28 (10/08/2021)	110.48 (17/11/2021)	6.69	0.44	Yes
	Finland	HIC	107.19 (10/08/2021)	148.36 (17/11/2021)	9.04	0.93	Yes
	France	HIC	115.67 (09/08/2021)	150.53 (16/11/2021)	11.26	0.68	Yes
	Germany	HIC	114.35 (10/08/2021)	138.5 (17/11/2021)	11.43	0.70	Yes
	Greece	HIC	104.34 (10/08/2021)	129.32 (17/11/2021)	7.72	0.59	Yes
	Hungary	HIC	108.50 (01/08/2021)	137.33 (15/11/2021)	6.70	100.00	Yes
	Iceland	HIC	139.84 (06/08/2021)	171.76 (16/11/2021)	9.47	178.00	Yes
	Ireland	HIC	124.65 (09/08/2021)	147.94 (16/11/2021)	6.93	1.13	Yes
	Italy	HIC	119.98 (10/08/2021)	153.68 (17/11/2021)	8.67	0.85	Yes
	Latvia	HIC	74.11 (10/08/2021)	113.60 (17/11/2021)	6.19	0.31	Yes
	Lithuania	HIC	104.20 (10/08/2021)	131.33 (17/11/2021)	6.57	0.30	No
	Luxembourg	HIC	117.52 (10/08/2021)	135.40 (14/11/2021)	5.29	0.99	Yes
	Malta	HIC	176.02 (09/08/2021)	177.28 (16/11/2021)	8.96	0.89	No
	Netherlands	HIC	121.54 (08/08/2021)	141.79 (15/11/2021)	9.97	0.89	Yes
	Norway	HIC	104.97 (09/08/2021)	146.42 (16/11/2021)	10.05	11.50	Yes
	Poland	HIC	92.78 (10/08/2021)	106.03 (16/11/2021)	6.33	1.30	No
	Portugal	HIC	126.92 (10/08/2021)	160.39 (15/11/2021)	9.41	0.55	Yes
	Romania	HIC	49.40 (09/08/2021)	73.06 (16/11/2021)	5.56	2.74	Yes
	Slovakia	HIC	79.88 (10/08/2021)	89.32 (16/11/2021)	6.69	0.35	Yes
	Slovenia	HIC	85.61 (10/08/2021)	113.04 (17/11/2021)	8.30	0.56	Yes
	Spain	HIC	127.11 (09/08/2021)	158.67 (16/11/2021)	8.98	0.74	Yes
	Sweden	HIC	110.33 (10/08/2021)	148.61 (17/11/2021)	10.90	11.20	Yes
	Switzerland	HIC	105.68 (09/08/2021)	130.91 (16/11/2021)	11.88	1.69	Yes
	United Kingdom	HIC	127.83 (09/08/2021)	161.58 (16/11/2021)	10.00	0.75	Yes
**Region of Americas**	Canada	HIC	134.72 (10/08/2021)	157.03 (17/11/2021)	10.79	1.22	Yes
	United States of America	HIC	105.41 (10/08/2021)	132.25 (17/11/2021)	16.89	1.27	Yes
**Western Pacific region**	Australia	HIC	54.74 (10/08/2021)	147.74 (17/11/2021)	9.28	1.47	Yes
	Japan	HIC	83.56 (10/08/2021)	154.96 (17/11/2021)	10.95	91.90	Yes
	New Zealand	HIC	47.56 (10/08/2021)	142.04 (17/11/2021)	9.21	1.08	Yes
**Economies in transition**							
**European region**	Albania	UMIC	43.78 (08/08/2021)	70.21 (16/11/2021)	5.26	23.50	No
	Armenia	UMIC	6.58 (08/08/2021)	28.03 (31/10/2021)	10.03	NA	Yes
	Azerbaijan	UMIC	54.03 (10/08/2021)	99.44 (17/11/2021)	3.51	NA	Yes
	Belarus	UMIC	25.19 (01/08/2021)	57.29 (07/11/2021)	5.64	NA	Yes
	Bosnia and Herzegovina	UMIC	23.26 (04/08/2021)	47.61 (04/11/2021)	8.90	0.59	Yes
	Georgia	UMIC	17.27 (10/08/2021)	52.12 (17/11/2021)	7.11	NA	No
	Kazakhstan	UMIC	54.61 (10/08/2021)	86.54 (17/11/2021)	2.92	NA	No
	Kyrgyzstan	LMIC	11.84 (09/08/2021)	28.67 (17/11/2021)	6.53	NA	No
	Montenegro	UMIC	55.63 (10/08/2021)	82.21 (16/11/2021)	8.42	0.64	No
	North Macedonia	UMIC	47.17 (09/08/2021)	79.60 (16/11/2021)	6.58	33.10	No
	Republic of Moldova	LMIC	27.56 (10/08/2021)	39.26 (17/11/2021)	6.60	NA	Yes
	Russian Federation	UMIC	46.14 (10/08/2021)	79.93 (17/11/2021)	5.32	39.60	Yes
	Serbia	UMIC	82.19 (09/08/2021)	108.87 (14/11/2021)	8.54	76.00	Yes
	Tajikistan	LIC	11.47 (04/08/2021)	49.77 (07/11/2021)	7.24	NA	No
	Turkmenistan	UMIC	NA	NA	6.61	NA	No
	Ukraine	LMIC	15.65 (10/08/2021)	50.18 (17/11/2021)	7.72	NA	Yes
	Uzbekistan	LMIC	24.80 (04/08/2021)	89.34 (17/11/2021)	5.29	NA	No
**Not among WHO member states**							
	Hong Kong SAR, China	HIC	84.24 (10/08/2021)	122.14 (17/11/2021)	NA	NA	NA
	State of Palestine	NA	20.37 (09/08/2021)	56.38 (10/11/2021)	NA	NA	NA
	Taiwan Province of China	HIC	38.90 (10/08/2021)	119.07 (17/11/2021)	NA	NA	NA

^¶^: Least developed countries;  : Heavily indebted poor countries. Abbreviations: COVID-19: Coronavirus disease-2019; HIC: High-income country; LIC: Low-income country; LMIC: Lower middle-income country; NA: Not available; UMIC: Upper middle-income country; USD: United States dollar; WHO: World Health Organization.

## Conclusion

After more than 50 years from the declaration of human rights, equitable accessibility (e.g., affordability and availability) to high-quality health services remains challenging in low-income, middle-income, and sanctioned regions. Due to people affliction in such regions, especially during the COVID-19 pandemic, this matter should be immediately addressed through identifying contributory factors (*i.e*., SDH, interregional and intraregional bodies), development of eliminating strategies, and establishing monitoring mechanisms to assess the impact of enacted policies on health outcomes and health services delivery. In sanctioned regions, global regulatory and welfare organizations have the leading role in confronting oppressive countries and maintaining health justice. Immediately tackling with lack of adequate funding for research on health disparities and the need for a faster data/evidence cycle for decision-making are other notable matters in the current crisis of global inequity. 

## Authors contribution

All authors made a significant contribution to the work reported, whether in the conception, study design, execution, acquisition of data, analysis, and interpretation, or in all these areas. That is, revising or critically reviewing the article; giving final approval of the version to be published; agreeing on the journal to which the article has been submitted; and confirming to be accountable for all aspects of the work. TM extracted the data and drafted the manuscript. SN and MA have drawn an idea and edited the paper.

## Declaration of Interests

The authors have no relevant affiliations or financial involvement with any organization or entity with a financial interest in or financial conflict with the subject matter or materials discussed in the manuscript. This includes employment, consultancies, honoraria, stock ownership or options, expert testimony, grants or patents received or pending, or royalties.

## Funding

The paper was not funded.
